# Draft Genome Sequence of a *Bordetella pertussis* Strain with the Virulence-Associated Allelic Variant *ptxP3*, Isolated in Italy

**DOI:** 10.1128/genomeA.00944-15

**Published:** 2015-09-10

**Authors:** A. Anselmo, G. Buttinelli, A. Ciammaruconi, F. Midulla, A. Nicolai, A. Fortunato, A. Palozzi, S. Fillo, F. Lista, P. Stefanelli

**Affiliations:** a Army Medical and Veterinary Research Center, Rome, Italy; b Department of Infectious, Parasitic and Immune-Mediated Diseases, Istituto Superiore di Sanità, Rome, Italy; c Department of Pediatrics, “Sapienza” University of Rome, Rome, Italy

## Abstract

Despite a universal immunization program, pertussis has persisted and resurged, and is of particular concern for infants in terms of morbidity and mortality. Here, we report the genome sequence of a *Bordetella pertussis* strain with the virulence-associated allelic variant *ptxP3*, isolated from a 45-day-old infant.

## GENOME ANNOUNCEMENT

Pertussis (whooping cough) is the most common vaccine-preventable disease, and despite widespread vaccination, the circulation of *Bordetella pertussis*, the causative agent of pertussis, has increased in many countries (1–3). One of the hallmarks of the resurgence of pertussis is the presence of polymorphisms in the virulence-associated genes, including the *ptx* promoter (*ptxP*); *ptxP3*, a novel allele for the pertussis toxin promoter, increases pertussis toxin production and is thus considered the most virulent (4). It replaced almost completely *ptxP1* among circulating strains and, together with the sequence analysis of other genes, is commonly evaluated for its genetic divergence from the vaccine strain (5).

Here, the draft genome sequence of a *B. pertussis* strain, isolated from an unvaccinated 45-day-old infant, is reported. The infant coughed for 33 days with paroxysms, and presented apnea and cyanosis. The strain was isolated from nasopharyngeal aspirate and lab-confirmed by routine conventional methods (6, 7). In particular, it showed the allelic profiles for *ptxA1*, *ptxP3*, *prn 2*, and MLVA 26.

Genomic DNA was extracted using the QIAamp DNA minikit (Qiagen, Hilden, Germany) from a culture grown on Charcoal Agar plates at 35°C. The library was prepared from the extracted genomic DNA using the Nextera XT DNA sample preparation kit (Illumina, San Diego, CA, USA), and a 2 × 300 nucleotide (nt) paired-end sequencing run was performed using the Illumina MiSeq platform. The reads were trimmed and *de novo* assembled using Abyss version 1.5.2 software (8). The assembly of 2,786,692 reads generated 299 contigs >500 nt with 235× coverage. The estimated genome size is 3.9 Mb, with a G+C content of 67.5%.

### Nucleotide sequence accession numbers. 

This whole-genome shotgun project has been deposited at DDBJ/EMBL/GenBank under the accession number LFUE00000000. The version described in this paper is the first version, LFUE01000000.
